# Controlled droplet transport to target on a high adhesion surface with multi-gradients

**DOI:** 10.1038/srep45687

**Published:** 2017-04-03

**Authors:** Siyan Deng, Weifeng Shang, Shile Feng, Shiping Zhu, Yan Xing, Dan Li, Yongping Hou, Yongmei Zheng

**Affiliations:** 1 Key Laboratory of Bio-Inspired Smart Interfacial Science and Technology of Ministry of Education, School of Chemistry and Environment, Beihang University, Beijing, 100191, China; 2 Department of Measurement Control and Information Technology, School of Instrumentation Science and Opto-electronics Engineering, Beihang University, Beijing, 100191, P. R. China

## Abstract

We introduce multi-gradients including Laplace pressure gradient, wettable gradient and wettable different gradient on a high adhesion surface via special wedge-pattern and improved anodic oxidation method. As a result of the cooperative effect mentioned above, controlled directional motion of a droplet on a high adhesion surface is realized, even when the surface is turned upside down. The droplet motion can be predicted and the movement distances can be controlled by simply adjusting the wedge angle and droplet volume. More interestingly, when Laplace pressure gradient is introduced on a V-shaped wettable gradient surface, two droplets can move toward one another as designed.

Controlled self-propelling of droplets has extensive application prospect in many fields^1^,^2^,^3^,^4^,^5^, such as microfluidic device, fog-harvesting, industrial filtration equipment and condensing apparatus^6^,^7^,^8^,^9^,^10^,^11^. It is well-known that in a microscale systems, surface tension overpowers the inertial force and becomes a dominant force^12^. Therefore, droplet actuation driven by wettable gradient has drawn much attention of researchers^13^. As reported, to drive a droplet on a wettable gradient surface, the contact angle hysteresis should be lower than 10° 14. Therefore, most current efforts are invariably limited to fabricate low adhesive surfaces^15^,^16^,^17^. For example, Sandra C *et al*. fabricated graphene surface with a well-controlled, chemical gradient to push the droplet motion in the direction of increasing hydrophilicity and low adhesion^18^. Yao *et al*. developed nanoporous elastic substrates coated in a lubricating liquid film to mitigate hysteresis force to manipulate a droplet on such surfaces^19^. Feng *et al*. constructed a low adhesion surface by anodic oxidation combined octafluorocyclobutane (C_4_F_8_) plasma to transport droplets accurately^20^. Most of low adhesive surface fabrication processes involve very elaborate design and complex processes. On the other hand, although controlled droplet movement on a high adhesion surface has important implications to both fundamental research and practical applications (medical test, biomedical domain and so on), less related work has been done due to high motion resistance. Therefore, it is urgent to resolve that how to simplify the process and reduce the usage restriction of wettable gradient surface using for self-propelling of droplets, i.e., not only on a low adhesive surface. To date, realizing the controlled directional motion of tiny water droplets on a high adhesion surface remains a great challenge. For example, even on a large wettable gradient surface (3.4°/mm)^21^, only directional spreading was observed due to high adhesive force.

Nature has given us some unbelievable ways of manipulating droplet^22^,^23^,^24^,^25^. For instance, spider silks utilize “periodic spindle knot” where chemical gradient and Laplace pressure are integrated to realize hanging droplets orientation movement^26^, which gives us a great example of self-propelling motion of droplets on a high adhesion surface. Inspired by cooperative effect on the surface of spider silks, here, we take one-step gradient oxidation method to form wettable gradient and then introduce Laplace pressure gradient via special wedge-patterns to fabricate wettable gradient and Laplace pressure gradient simultaneously on a high adhesion surface. By the collaborative effect of two gradients, spontaneous pumpless directional motion of micro-sized droplet on a high adhesion surface can be achieved, even when the surface is turned upside down. And movement distance of droplet can be easily controlled by adjusting the wedge angle and droplet volume. More interestingly, two droplets can move to one another and merge into one as designed on a surface with V-shaped wettable gradient and Laplace pressure gradient. This study provides a novel insight into a wettable gradient surface for the micro-droplet motion in directions on the high adhesion surface, which is significant for designing smart materials that can be extended to the realm of controlling fluidic transport in directions, pharmaceutical detection, microfluidic tools and so on.

## Results and Discussion

**Figure 1:**
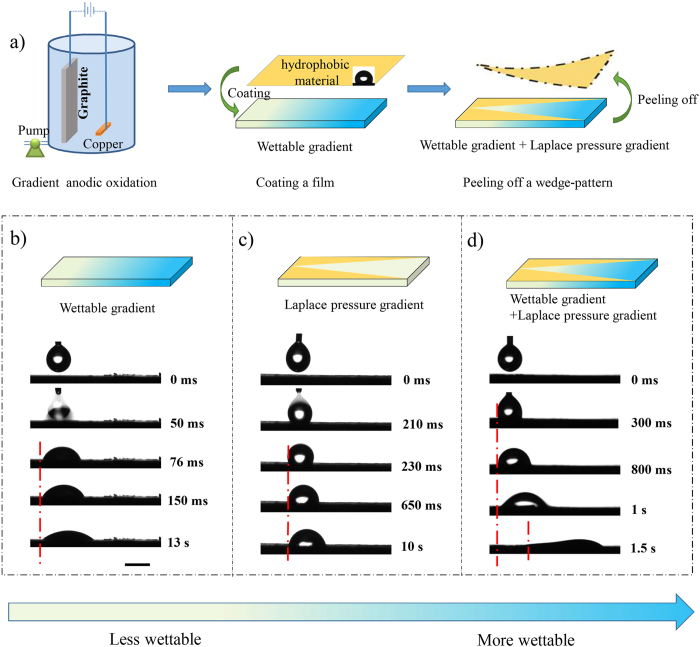
(**a**) Schematic illustration of the formation of wettable gradient and Laplace pressure on a high adhesion surface. Treating graphite plate via gradient anodic oxidation to form wettable gradient; coating the prepared graphite plate with hydrophobic material to ensure a hydrophobic surrounding; peeling off a wedge-pattern to induce Laplace pressure gradient on the wettable gradient surface. (**b**) Droplet movement behaviour on surface with wettable gradient. (**c**) Droplet movement behaviour on surface with wedge-pattern. (**d**) Droplet movement behaviour on surface with wettable gradient and wedge-pattern. The droplets can be driven only on the surface combining wettable gradient and Laplace pressure. The scale bar is 2 mm.

Liquid transport on wedge-pattern surfaces was first studied by Khoo and Tseng. They presented liquid transport on nanotextured surface with wedge-shaped track^27^. However, the fabrication process was complex and droplet movement distance was uncontrolled as the wedge angle was too small to control precisely. Alheshibr *et al*. demonstrated droplet spontaneous droplet movement on hydrophilic aluminium surface containing a hydrophobic background^28^. Although droplet spreading distance could be controlled through the change in wedge angle, the droplets tended to spread instead of moving. Here, in order to realize the controlled self-propelling of droplet on a high adhesion surface (droplet can’t slide even when the plate is placed vertically), we combine wettable gradient and Laplace pressure simultaneously on a surface. Surfaces with wettable gradient and Laplace pressure gradient were fabricated as shown in Fig. 1a. At first, graphite plates were prepared by gradient anodic oxidation to form wettable gradient via the change in the surface chemical composition (see Figure S1), as our previous report^21^. The difference of SEM image in different regions is not obvious (see Figure S2), from which we can conclude that the wettability gradient is caused by the difference of chemical composition. By controlling the volume flow of electrolyte, the wettable gradient can be controlled between 1.2°/mm and 9°/mm, as shown in Figure S3. Then, the prepared graphite plates were coated with paraffin wax to ensure a hydrophobic background (the coated substrate exhibits average sessile contact angle (CA) of 116°), and a wedge-pattern area was removed to induce Laplace pressure gradient on the wettable gradient surface. The SEM image shows distinct wedge-pattern and clear dividing line, whereas the roughness between covered portion and uncovered portion is similar (see Figure S4). This is a simple but effective way to produce wettable gradient and Laplace pressure gradient simultaneously on a surface of conductive substrates such as carbon and some metal materials. The wettable gradient and Laplace pressure gradient can be adjusted via oxidation conditions and wedge angle, respectively. Here, we choose the graphite surface with a wettable gradient of 9.0°/mm (the change of CAs is shown in Figure S5) to investigate the movement behaviours of droplets on it.

In order to present the necessity of the combination of a wettable gradient and a wedge-pattern, experiments of the graphite plates only with wettable gradient or with wedge-pattern were carried out comparing with the sample with wettable gradient and wedge-pattern simultaneously. As shown in Fig. 1b, longitudinal spreading can be observed on the surface with wettable gradient due to the existence of the gradient force (the wettable gradient is 9.0°/mm). Droplet on the sample with wedge-pattern also spreads a little bit as the result of Laplace pressure (wedge angle is 20°) (Fig. 1c). Clearly, droplets can not move, whether on the sample with wettable gradient or wedge-pattern. However, with the combination of wettable gradient and wedge-pattern (wedge angle is 20° and wettable gradient is 9.0°/mm) (see Fig. 1d), droplet moves unidirectionally as designed, from which we can confirm the necessity of combining wettable gradient and wedge-pattern to drive droplets.

**Figure 2:**
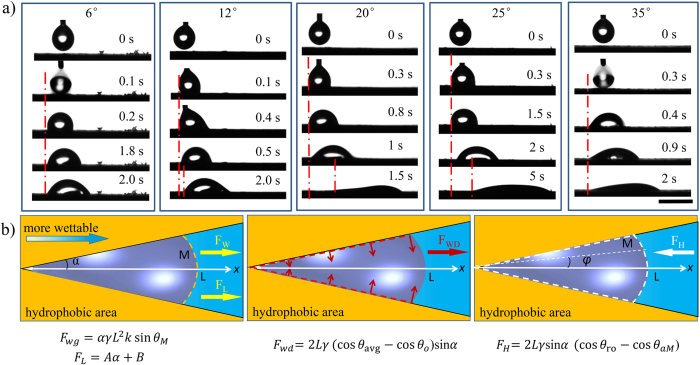
(**a**) The droplet movement behaviour on the graphite plates with different wedge-patterns. (**b**) The analysis of forces acted on the droplet, i.e., cooperation of the three driving force: wettable gradient force (*F*_*wg*_), Laplace pressure force (*F*_*L*_) and wettability different force (*F*_*wd*_). The scale bar is 3 mm.

The wedge angle is one of the most critical influences on the droplet motion^29^. The movement behaviour of water droplet of 5 μL on the wettable gradient surfaces (9.0°/mm) with different wedge angles is shown in Figs 2a and S6. When we add a wedge angle of 6° on wettable gradient surface, droplet stays still due to less Laplace pressure gradient. Even the angle increases to 10°, droplet remains still. When the angle is between 12° and 32°, the obvious self-propelling of droplets is observed and the movement distance gradually increases at first and then decreases remarkably, showing a peak of 2.6 mm when the angle is 20° (Figure S7). When the angle is above 35° (including 35°), droplet just spreads along the direction of wettable gradient and no movement behaviour is observed. Figure S8 shows pictures of 5 μL droplet as it moves on the surface with wedge pattern of 20°. The droplet driven by the wettable gradient and Laplace pressure moves along the wedge track. The droplet evolves from a sphere droplet into cone shape. Clearly, we realize the self-propelling of droplets on a high adhesion surface via the collaborative effect of Laplace pressure and wettable gradient and the movement distance can be adjusted by the angle of wedge-patterns.

For a thorough understanding of the movement behaviour of water droplets on surfaces with wettable gradient and Laplace pressure, we analyze the forces exerted on the droplet (Fig. 2b). The droplet motion on the gradient surface involves the interaction effect between the driving forces and resistance force. The driving forces consist of wettable gradient force (*F*_*wg*_), Laplace pressure force (*F*_*L*_) and wettable different force (*F*_*wd*_). The resistance force is hysteresis force (*F*_*H*_). On wettable gradient surface, a driving force *F*_*wg*_ arising from the wettable gradient, due to different CAs at the both ends of the droplet, can be described by refs 21 and 26:


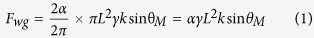


In which *α* is the half wedge angle, *γ* is the surface tension of water, *L* is the length of droplet along the wettable gradient direction and *k* is the wettable gradient (here, *k* = 9°/mm) and *θ*_*M*_ is the CA at more wettable side (M) of droplet.

Due to wedge-pattern on wettable gradient surface, Laplace pressure force (*F*_*L*_) in the lengthwise direction is generated, which push the droplet from a smaller wettable footprint (left) to a larger one (right). Megaridis *et al*.^30^ followed the approach of Lorenceau and Quéré^31^ to calculate the Laplace pressure force (*F*_*L*_) on the droplet sat on the wedge-pattern and found out the linear nature of the plot of *F*_*L*_ against *α*. Accordingly, we can evaluate the magnitude of the *F*_*L*_ as:


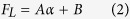


here, *A, B* is constant coefficient for a given volume of droplet.

In addition, the difference of wettability between inside and outside of wedge-pattern also provides another driving force (*F*_*wd*_) in the *x* direction, which can be described by ref. 26:


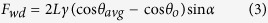


In which *θ*_*avg*_ is the average contact angle of droplet over the length of the drop and *θ*_*o*_ is the contact angle of hydrophobic area outside of wedge-pattern. Here, we use simple method to introduce three driving forces on a high adhesion surface, i.e., wettable gradient force, Laplace pressure force and wettability different force (Fig. 2b).

The Hysteresis force, which is always opposite to the moving direction, is composed of three parts (an arc and two straight-line edges)^21^:


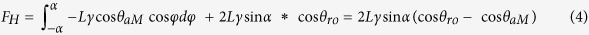


Here *θ*_*aM*_ is the advancing CA at more wettable side of water, *φ* is the polar angle and *θ*_*ro*_ is the apparent receding CA of hydrophobic area outside of wedge-pattern (*θ*_*ro*_* = *105°).

Obviously, there seem to be four main forces influencing the motion of droplets: wettable gradient force (*F*_*wg*_), Laplace pressure force (*F*_*L*_), wettable different force (*F*_*wd*_) and hysteresis force (*F*_*H*_). On the basis of the equations (1), (2), (3) and (4), the total force (*F*_*T*_) is described as:


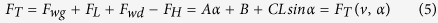


Here, 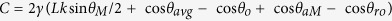. For a given volume of droplet (*v*), the values of *A, B, C* and *L* can be seen as constant. Therefore, total force (*F*_*T*_) can be regarded as a function of the volume of droplet (*v*) and the half wedge angle (*α*). For a given volume, we can measure the value of the length of droplet (*L*). Then, according to the Figure S9, the values of *θ*_*M*_, *θ*_*avg*_, *θ*_*aM*_ can be read right off the diagram. So, the value of *C* can be obtained by calculating. Finally, we observe the motion behaviors of droplet at different wedge angles. Accoridng to the critical condition under which droplets can self-propel, we can get the values of *A* and *B* for given volume of droplet.

For example, for droplet of 5 μL, the contact length *L* is 2.24 mm. From Figure S9, We can get the the values of *θ*_*M*_, *θ*_*avg*_, *θ*_*aM*_, i.e., 93°, 103.3°, 112.6°. Accordingly, we can figure out the value of *C (C* = 0.008749) easily. For droplet of 5 μL, the droplet can self-propell when the wedge angle is between 12° and 32°, i.e., *F*_*T*_(6°) = *F*_*T*_(16°) = 0. So, we can get Values of *A* = −0.000082689 and *B* = 0.00000015826. By the same method, the values of *A, B, C* for droplets of 2 μL, 8 μL, 10 μL can be achieved by calculating and the results are list in Table 1.

**Figure 3:**
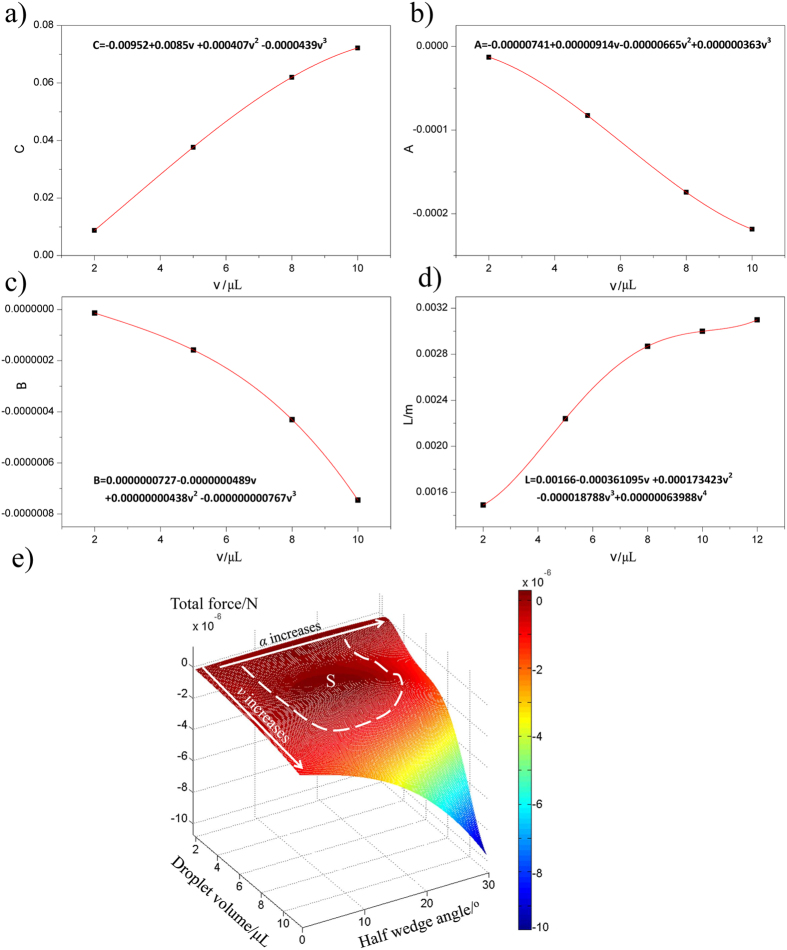
(**a**–**d**) The fitted function (*C, A, B and L*) versus volume of droplet (*v*) respectively according to polynomial equations. (**e**) Total force exerted on the droplet versus the droplet volume and half wedge angle.

As metioned in the previous part, the *A, B, C* and *L* are function of volume (*v*), so we use polynomial equations to get the relations between *A, B, C, L and v*, as shown in Fig. 3a–d. So, we could further simplify express the equation of total force (*F*_*T*_):


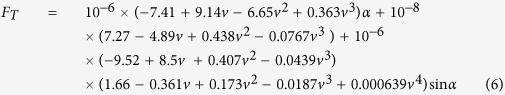


According to equation (6), a mathematical model based on an analysis of the forces applied on liquid drops is set up (The force acted on the droplet is determined by the droplet volume (*v*) and half pattern wedge angle (*α*)). As shown in Fig. 3e, the movement behaviour of droplets may be determined from the value *F*_*T*_ and the conditions, under which the value *F*_*T*_ is above zero, are marked (area S). The results indicate that actuation range reduces monotonically with droplet volume, which matches well with our experiment results listed in the Table 1. Otherwise, as can be seen from the simulation diagram, when the droplet volume is around 6.3 μL, the driving force is the largest at certain wedge angle. And when the droplet volume reaches about 11 μL, the driving force reduces to zero at all wedge angle. Besides that, as shown in the graph, when the droplet volume is fixed, the force applied on the droplet increases firstly and then decreases with the increase of wedge angle, showing a peak value. All of results are accorded with observation results, i.e., the droplet exhibits its maximum average motion velocity when the volume is 6.5 μL (in Figure S10). When droplets volume reaches 11 μL, the droplet can not be driven at any wedge angle. The movement distance reaches its peak value when the half wedge angle is 10° for 5 μL droplet (Figure S7). Not unexpected, the simulation result corresponds well with our experimental result, which revealed the validity of numerical simulation. Clearly, we can predict droplet movement behaviour via the droplet volume (*v*) and half pattern wedge angle (*α*) (Fig. 3e), and to control the droplets motion by adjusting the droplet volume and wedge angle. The optimized wedge angle is depend on the droplet volume, and the optimized amount droplet volume can be discussed in two aspects. One is the amount of 2 uL which can be actuated among the widest range of wedge angle (from 7° to 30°). The other is the amount of 5 uL which exhibited the longest motion distance among the maximum movement distance of different droplet volume (see Figure S11).

**Figure 4:**
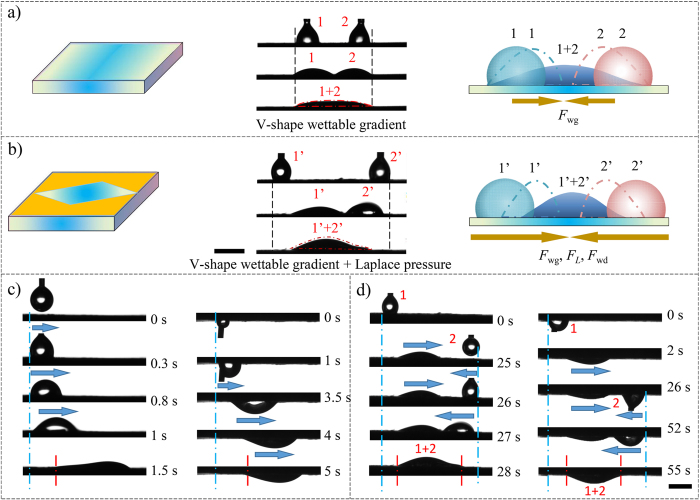
(**a**) Droplet movement behaviours on the graphite plate with V-shape wettable gradient and corresponding force analysis. Two separate droplets (marked as 1, 2) can spread closely to each other and merge into one if the distance between two droplets is short. The driving force arises from wettable gradient force (*F*_*wg*_). (**b**) Droplet movement behaviours on the graphite plate with V-shape wettable gradient and rhombus-shape pattern and corresponding force analysis. Two droplets (marked as 1′, 2′) move toward to each other and then merge quickly into one even the distance between two droplets is much longer, which is due to the cooperation of three driving forces, i.e., wettable gradient force (*F*_*wg*_), Laplace pressure force (*F*_*L*_) and wettable different force (*F*_*wd*_). (**c**) Water droplet movement behaviours on a surface with unidirectional gradients and Laplace pressure. (**d**) Water droplet movement behaviours on a surface with V-shaped gradients and Laplace pressure. The results show that a droplet can move unidirectionally on a surface with unidirectional gradients and two water droplets (marked as 1, 2) move towards each other and merge into one on a surface with V-shaped gradients, even when the as-prepared surface is turned upside down. The scale bar is 2 mm.

Apart from the single droplet motion, we also fabricated a surface with V-shape wettable gradient and Laplace pressure to realize the controlled motion of two droplets on the basis of the aforementioned method. Firstly, we took a one-step method to form V-shape gradient. Next, the graphite plate was coated by the wax and a rhombus area was removed. By doing so, V-shape wettable gradient and Laplace pressure gradient are formed on one surface. Figure S12 shows that both ends of the graphite plate have CAs about 108° as the oxidation time is the shortest. The smaller the distance between the measurement point and the centre, the more hydrophilic the position exhibited. i.e., CAs change gradually from 108° to 31° along the direction from the boundary to the centre. As shown in Figure S13, we can see apparent rhombus pattern and the dividing line is distinct in the optical images. In order to observe the movement behaviour of two droplets within limited field of vision, here we chose 2 μL droplet in the following experiment. As shown in Fig. 4a, on the surface only with a V-shape wettable gradient, two separate droplets placed on the either side of the centre spread closely to each other and merge into one if the distance between two droplets is short (below 3.5 mm), while orientation movement of whole droplets can’t be seen. Here, by one-step gradient oxidation method, we can realize unidirectional spreading of two droplets and two droplets spread toward each other and merge into one droplet if the distance is short. The main driving forces arise from wettable gradient alone. However, once the distance of the droplets is above the critical distance (3.5 mm), droplets can’t merge into one (see Figure S14). After Laplace pressure is introduced via rhombus-pattern, the movement behaviour of droplets is different. Two droplets move toward to each other and then merge quickly into one (Fig. 4b). Both of two droplets move about 1.5 mm before merging into one due to the cooperation of three driving forces: wettable gradient force, Laplace pressure and wettability different force, as mentioned above. Because of the movement behaviour, two droplets can merged into one droplet even when they are placed farther apart (7 mm). The critical distance that droplets can merge into one is much larger than that on the surface merely of V-shape wettable gradient. This phenomenon is very important for micro reactor. By adjusting the angle and the volume of droplets, as well we can control the movement distance of the two droplets. On this basis, efforts are currently underway to fabricate more interesting pattern to realize individual or multiple droplets mobilization as design.

In addition, due to the high adhesion of graphite plate, even when the graphite plates are inverted, similar movement behaviour can be observed, as shown in Fig. 4c,d. Comparing movement behaviour of droplets in different conditions, when the plates are inverted, the profile of the droplet is more obvious due to the gravitational force and movement distance of droplets on the lower surface is longer than that on upper surface, which may be due to the fact that adhesion force is reduced under effect of gravity.

In conclusion, by combing wettable gradient and Laplace pressure gradient, unidirectional or bidirectional movement of droplets on a high adhesive surface can be realized, even when the surface is turned upside down. More interestingly, the movement distance can be controlled easily via adjusting the angle of the wedge-pattern. On this basis, more interesting shape pattern could be designed to manipulate individual droplet or multiple droplets, which has great potential application prospect in pharmaceutical detection and microfluidic tools^32^,^33^,^34^.

## Methods

### Fabrication of graphite plates with wettable gradient and Laplace pressure

A graphite plate (size of 30 × 10 × 2 mm^3^) with wettable gradient was obtained by gradient anodic oxidation treatment as previously reported. Then the graphite was coated with paraffin wax to fabricate a hydrophobic background and a wedge-pattern was removed to induce Laplace pressure gradient.

### Preparation of a graphite plate with V-shape wettable gradient and Laplace pressure

The V-shape wettable gradient was formed by one-step method. As shown in Figure S15, a narrow copper plate was used as cathode which was parallel to the graphite plate center. The lower part of the graphite was immersed in the oil, and the upper part was in the electrolyte (the density of the oil was much higher than that of the electrolyte). The interface between oil and water was flush with the graphite plate center. A hole in the bottom was used to run off the oil until the electrolyte level reached graphite center. After that, the graphite plate was coated with wax and a rhombus area was removed to introduce Laplace pressure (the diagonal line of rhombus coincided with the central line of the graphite plate).

### Characterization

Water contact angles (CAs) and droplet directional movement behaviours are recorded by the optical contact angle meter system (OCA40Micro, Dataphysics Instruments GmbH, Germany). A 5.0 μL droplet of deionized water is dripped upon the graphite plate to measure the droplet CAs and the static CAs are determined as the average of at least five measurements. The droplet movement behaviours are also recorded at least three times.

## Additional Information

**How to cite this article:** Deng, S. *et al*. Controlled droplet transport to target on a high adhesion surface with multi-gradients. *Sci. Rep.* **7**, 45687; doi: 10.1038/srep45687 (2017).

**Publisher's note:** Springer Nature remains neutral with regard to jurisdictional claims in published maps and institutional affiliations.

## Supplementary Material

Supplementary Information

## Figures and Tables

**Table 1:** Actuation range and the calculated values of *L, C, A, B* for different volumes of droplets.

Droplet volume [μL]	Actuation range (wedge angle)	*L*	*C*	*A*	*B*
2	7°–30°	1.49 × 10^−3^	8.75 × 10^−3^	−1.284 × 10^−5^	−1.37769 × 10^−8^
5	12°–32°	2.24 × 10^−3^	0.03764	−8.2689 × 10^−5^	−1.5826 × 10^−7^
8	15°–30°	2.87 × 10^−3^	0.06199	−1.74327 × 10^−4^	−4.3084 × 10^−7^
10	20°–30°	3 × 10^−3^	0.07219	−2.1849 × 10^−4^	−7.4508 × 10^−7^
