# Supplementary material for: Controlled droplet transport to target on a high adhesion surface with multi-gradients
Source: Sci Rep. 2017 Apr 3;7:45687. doi: 10.1038/srep45687 (PMC5377364; doi:10.1038/srep45687)
Supplement: Supplementary Information [file srep45687-s1.pdf]

# Supplementary Information

**Controlled droplet transport to target on a high adhesion surface with multi-gradients**

*Siyan Deng, Weifeng Shang, Shile Feng, Shiping Zhu, Yan Xing, Dan Li, Yongping Hou* and Yongmei Zheng**

[*] Prof. Y. Zheng, Prof. Y. Hou, S. Deng, W. Shang, Dr. S. Feng, Y. Xin, D. Li

Key Laboratory of Bio-Inspired Smart Interfacial Science and Technology of Ministry of Education, Beijing Key Laboratory of Bio-inspired Energy Materials and Devices School of Chemistry and Environment
Beihang University
Beijing, 100191 (P. R. China)
E-mail: houyongping09@buaa.edu.cn, zhengym@buaa.edu.cn

Prof. S. Zhu
Department of Measurement Control and Information Technology, School of Instrumentation Science and Opto-electronics Engineering,
Beihang University,
Beijing, 100191 (P. R. China)

## Content:

Supplementary Figure Legends: Figure S1-S15

Supplementary Figure Legends：

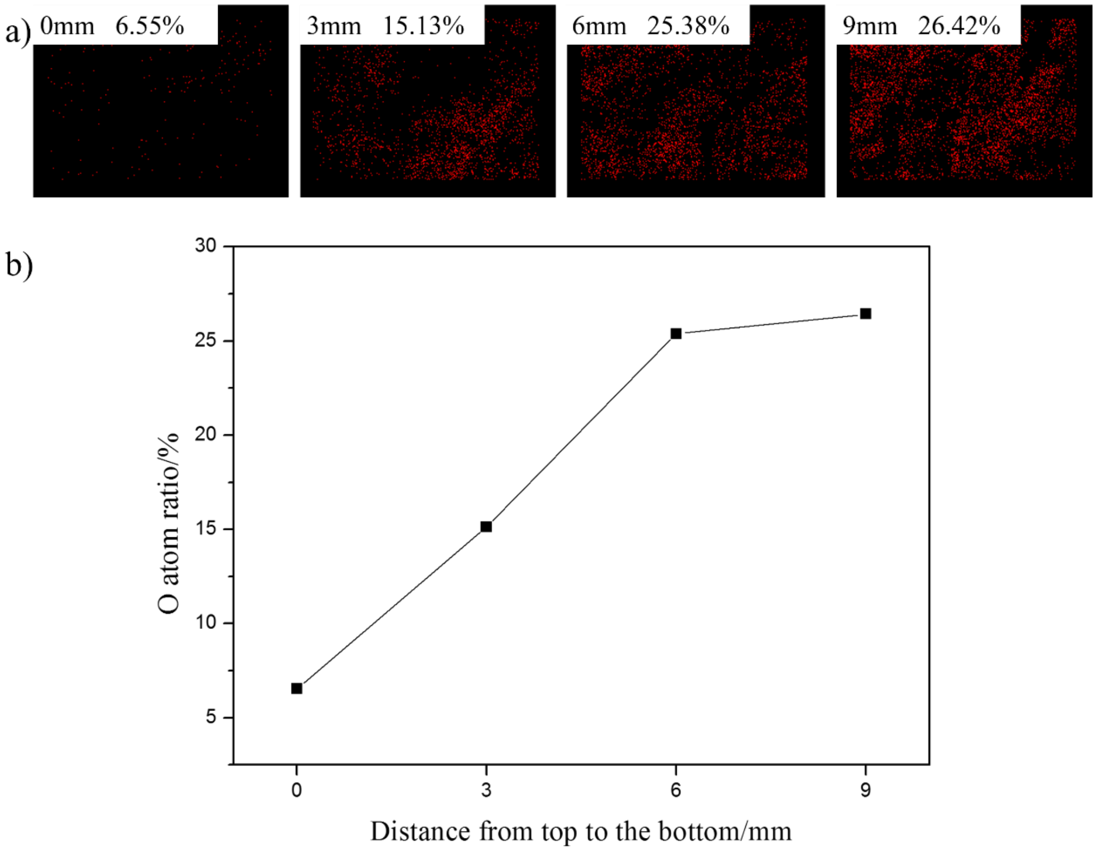


**Figure S1. O atom ratio at the different points. a)** O atom ratio of different points on the oxidized graphite plates (distance from the top to the bottom). **b)** The relationship between O atom ratio and the position of the oxidized graphite plate.

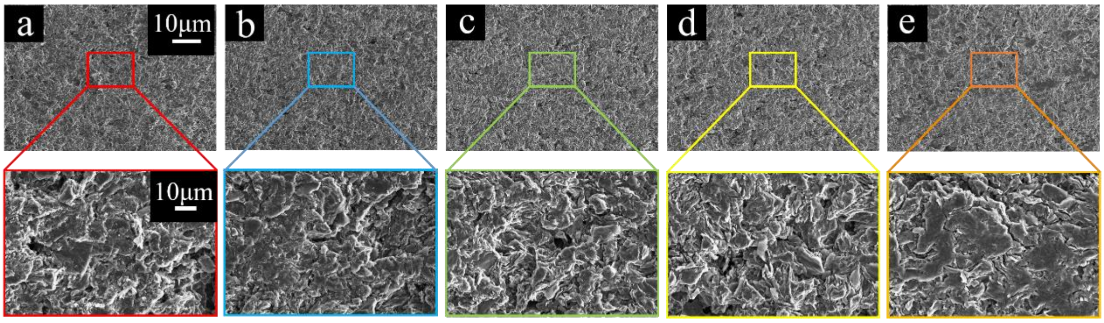


**Figure S2. SEM image of anodic graphite plate at different areas. a)** 2 mm. **b)** 4mm. **c)** 6 mm. **d)** 8 mm. **e)** 10 mm (distance from the top to the bottom). The scale bar is 10 μm.

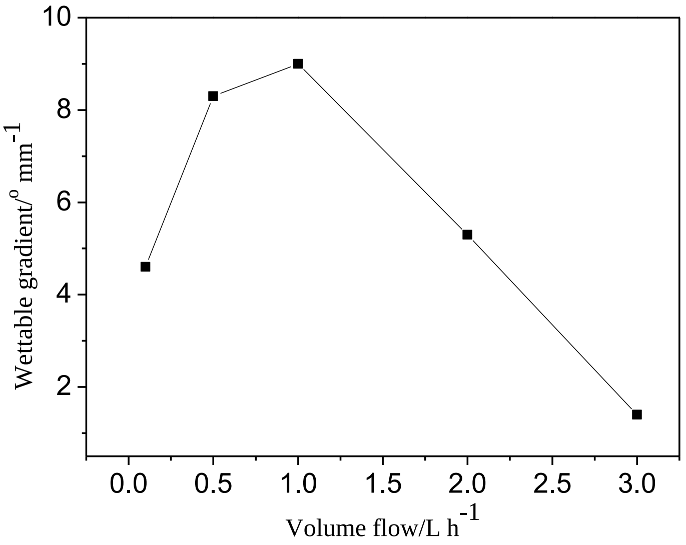


**Figure S3. Relationship between wettable gradient and electrolyte volume flow.** By controlling volume flow, we can adjust wettable gradient from 1.2$^{o}$/mm to 9$^{o}$/mm. With the increase of volume flow, wettable gradient increase first and then decrease remarkably, showing a peak of 9$^{o}$/mm as the volume flow is 1.0 L /h.

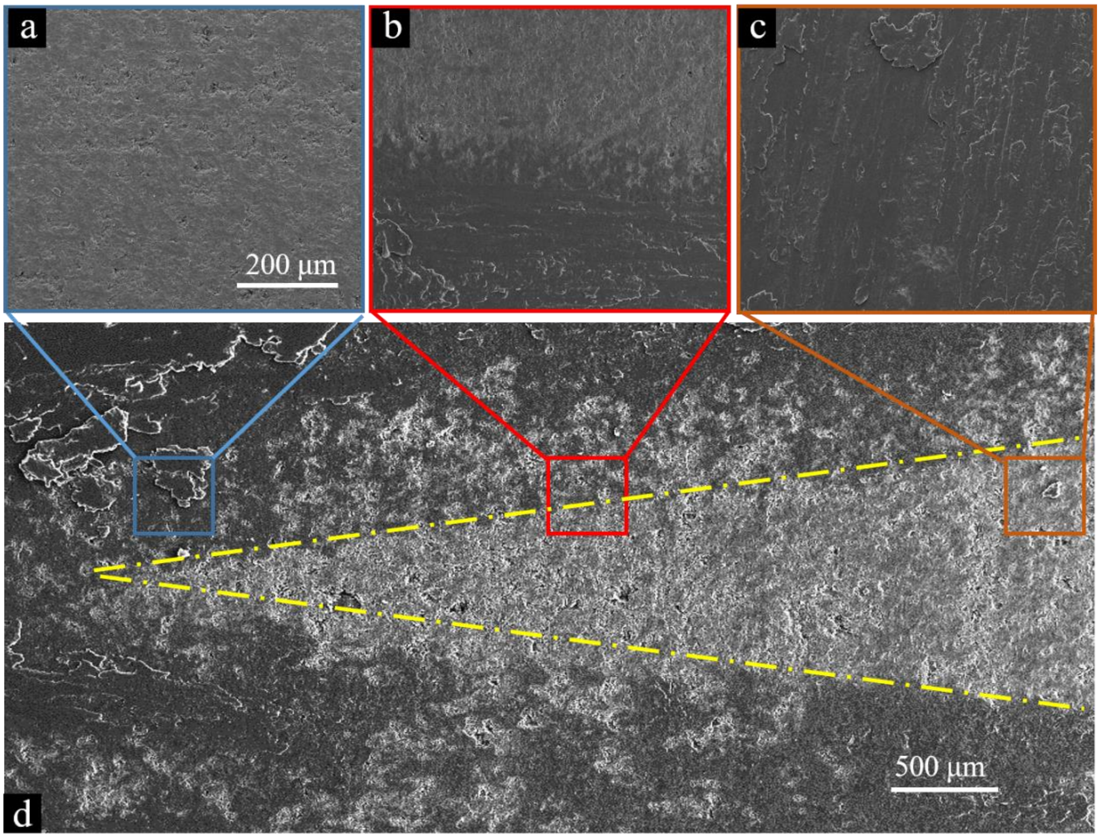


**Figure S4. SEM images of the wax-covered anodic graphite plate at different areas. a)** The wax-covered area, **b)** The boundary of wax-covered area and uncovered area, **c)** The uncovered area. **d)** The whole wedge pattern.

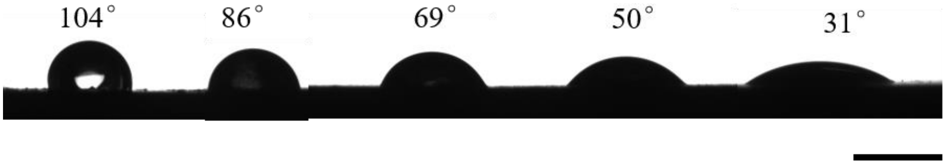


**Figure S5. The water contact angle along the wettable gradient surface.** The graphite plate is treated at a constant current of 0.5 A and the volume flow is 1 L/h. Clearly, wettable gradient is formed after gradient oxidation treatment. The scale bar is 1mm.

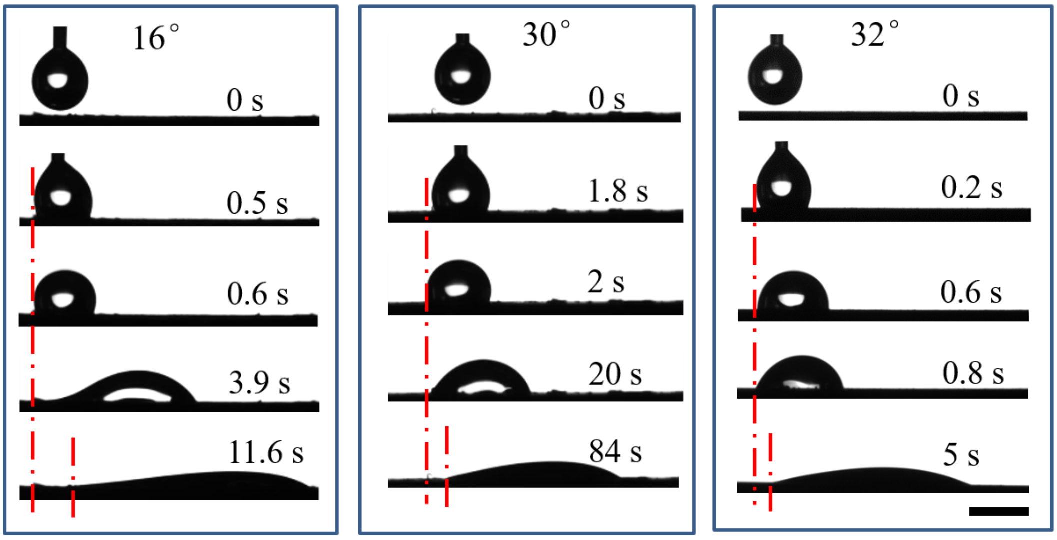


**Figure S6. The droplets movement behaviours on the oxidized graphite plates with different wedge angles (16º, 30º, 32º).** The droplet volume is 5 μL and the scale bar is 2mm.

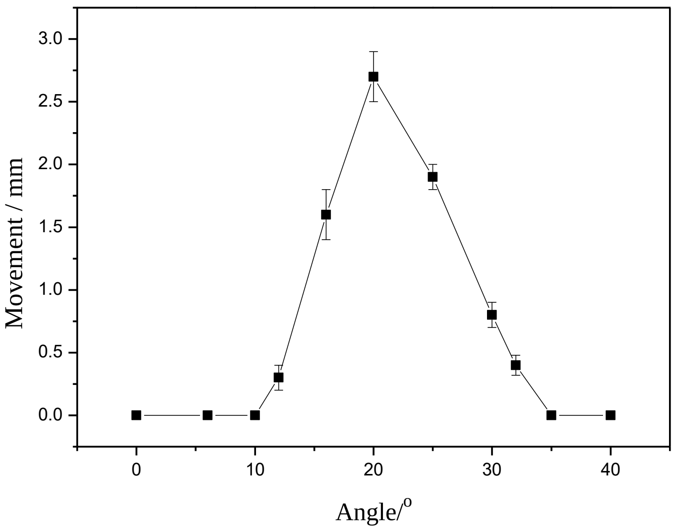


**Figure S7. Relationship between movement distance and the wedge angle.** With an increase in the wedge angle, the movement distance increases first and then decreases remarkably, showing a peak of 2.6 mm when the wedge angle is 20°.The volume of the droplet is 5 μL.

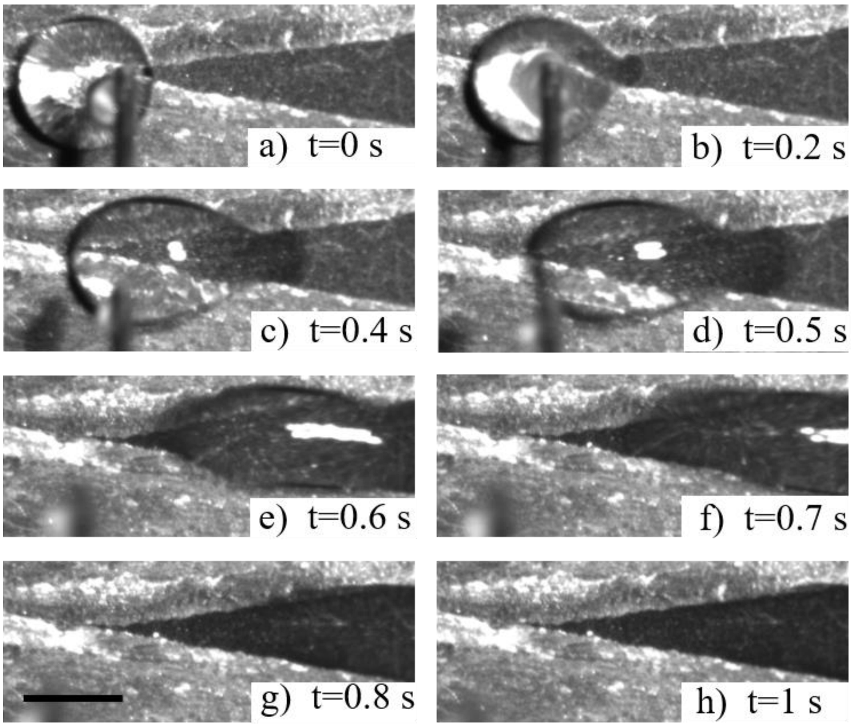


**Figure S8. Top view of droplet motion on the surface of wedge pattern.** The volume of the droplet is 5 μL and the wedge angle is 20$^o$. The droplet moves along the wedge track and the droplet evolves from a sphere droplet to cone shape. The scale bar is 2mm.

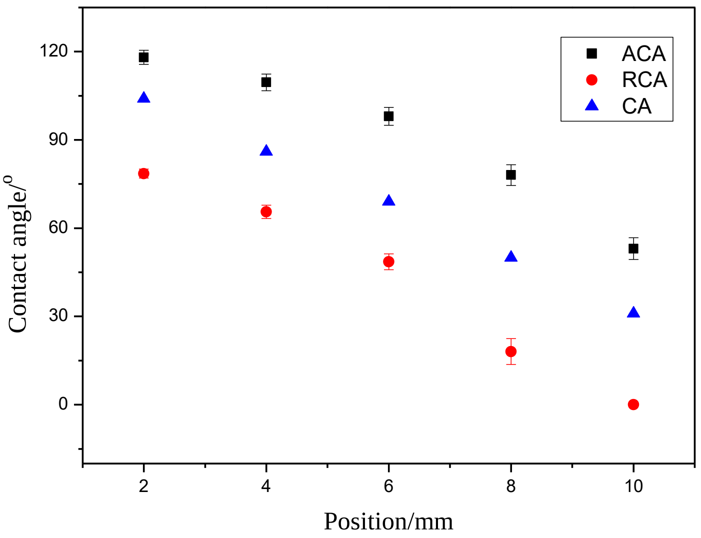


**Figure S9. The advancing CAs, receding CAs and CAs at the different points (distance from the top to the bottom).**

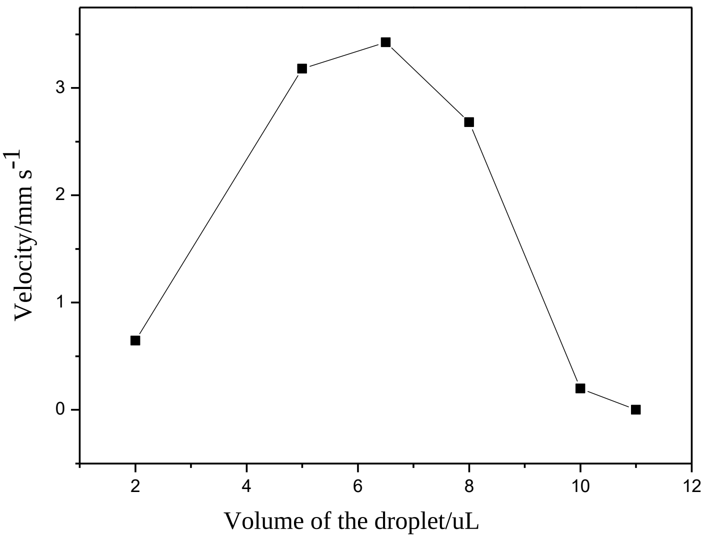


**Figure S10. The droplet velocity of movement versus the volume of the droplet.** With the increase of volume, the velocity increases first and then decreases remarkably, showing a peak of 3.5 mm/s (the wedge angle is 20$^o$).

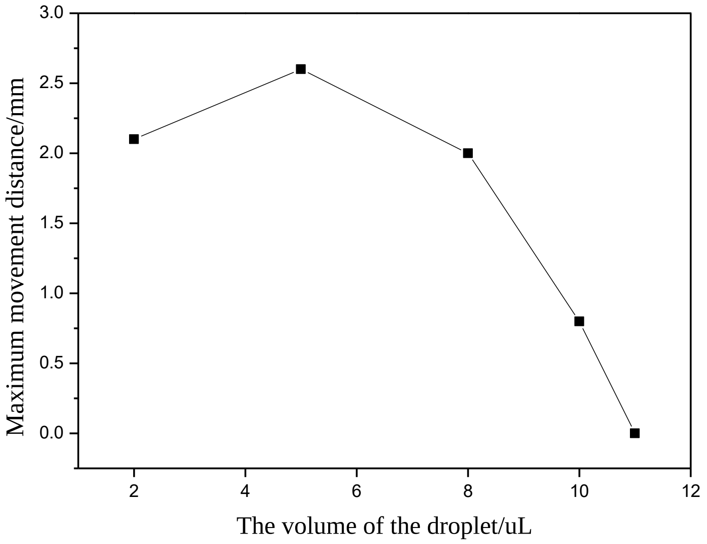


**Figure S11. The maximum movement distance versus the volume of the droplet.** The amount of 5 μL which exhibited the longest motion distance among the maximum movement distance of different droplet volume

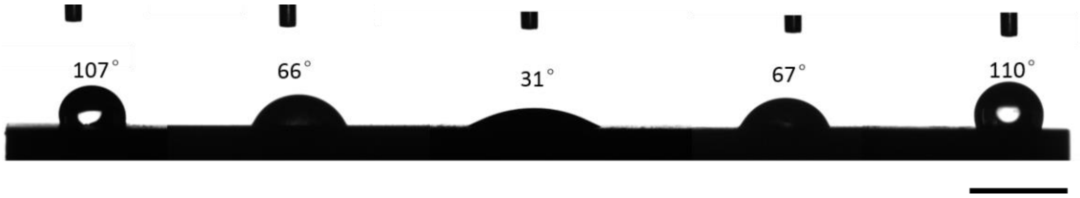


**Figure S12. Contact angles on V-shape wettable gradient surface.** The V-shape wettable gradient is successfully fabricated by one-step method mentioned. The graphite plate is treated at a constant current of 0.5 A and the volume flow is 0.5 L /h. The scale bar is 1 mm.

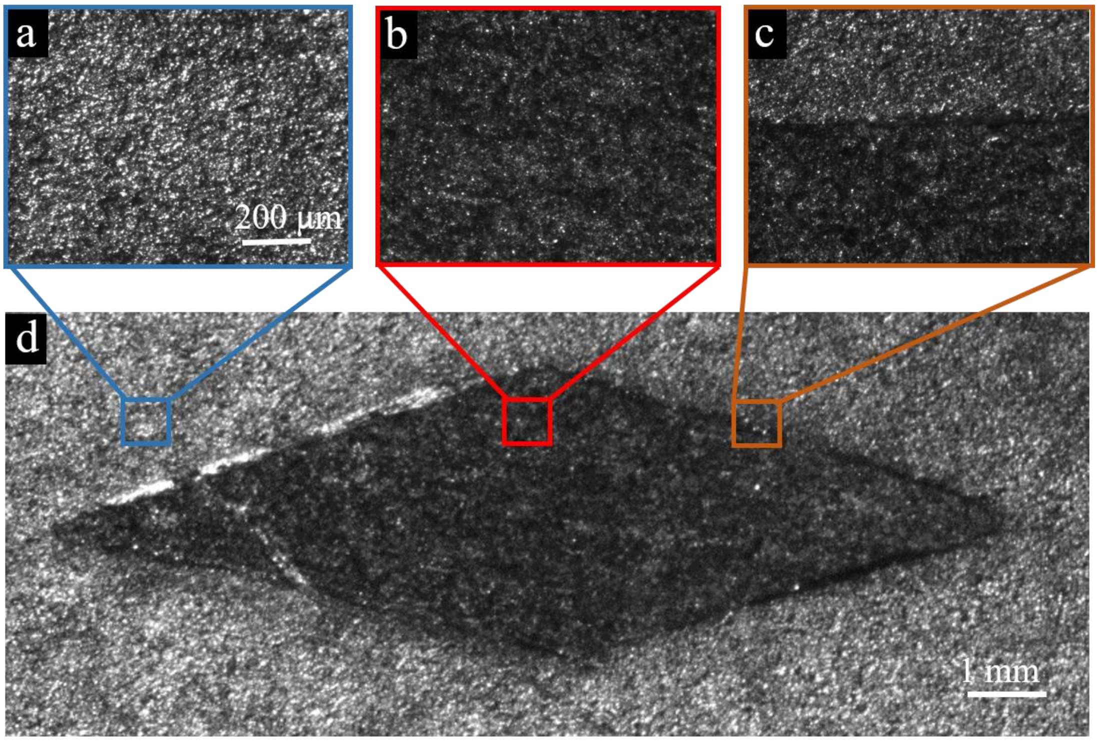


**Figure S13. Optical images of V shape structure. a)** The wax-covered area, **b)** The uncovered area. **c)** The boundary of wax-covered area and uncovered area, **d)** The whole rhombus pattern.

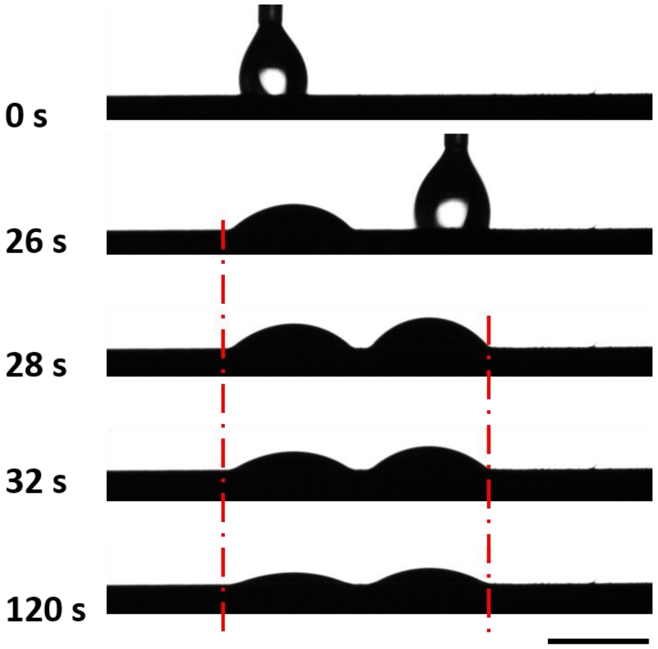


**Figure S14. Movement behaviour of droplet on the surface merely of V-shape wettable gradient (the distance between droplets is 3.8 mm).** It shows that droplets can not merge into one when the distance between droplets is above the critical distance (3.5 mm). The scale bar is 2 mm.

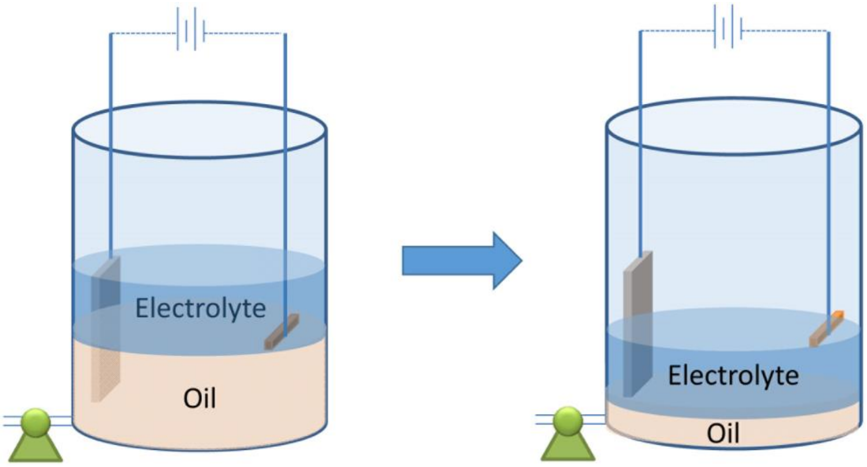


**Figure S15. Schematic illustration of the formation of V-shape gradient on the graphite plates.** During the oxidation process, a narrow copper plate was used as cathode, parallel to the transversal central line of the graphite plate. The lower part of graphite plate was immersed into the oil and upper part was in electrolyte. The interface between oil and water was flush with the transversal central line of the graphite plate. Once the oxidation reaction began, valve was opened to run off the oil to form oxidation time gradient. When electrolyte level reached the central line of the graphite, the oxidation finished.
